# Treatment outcomes of tuberculous meningitis in adults: a systematic review and meta-analysis

**DOI:** 10.1186/s12890-019-0966-8

**Published:** 2019-11-06

**Authors:** Ming-Gui Wang, Lan Luo, Yunxia Zhang, Xiangming Liu, Lin Liu, Jian-Qing He

**Affiliations:** 1 Department of Respiratory and Critical Care Medicine West China Hospital, Sichuan University, No. 37, Guo Xue Alley, Chengdu, 610041 China; 2 Chengdu Medical College, Chengdu, Sichuan Province People’s Republic of China; 3 Department of Respiratory and Critical Care Medicine, 363 Hospital, Chengdu, Sichuan Province People’s Republic of China

**Keywords:** Tuberculous meningitis,, Outcomes,, Adults,, HIV

## Abstract

**Background:**

Tuberculous meningitis is the most devastating presentation of disease with *Mycobacterium tuberculosis*. We sought to evaluate treatment outcomes for adult patients with this disease.

**Methods:**

The Ovid MEDLINE, EMBASE, Cochrane Library and Web of Science databases were searched to identify all relevant studies. We pooled appropriate data to estimate treatment outcomes at the end of treatment and follow-up.

**Results:**

Among the articles identified, 22 met our inclusion criteria, with 2437 patients. In a pooled analysis, the risk of death was 24.7% (95%CI: 18.7–31.9). The risk of neurological sequelae among survivors was 50.9% (95%CI: 40.2–61.5). Patients diagnosed in stage III or human immunodeficiency virus (HIV) positive were significantly more likely to die (64.8, 53.4% respectively) during treatment. The frequency of cerebrospinal fluid (CSF) acid-fast-bacilli smear positivity was 10.0% (95% CI 5.5–17.6), 23.8% (15.2–35.3) for CSF culture positivity, and 22.3% (17.8–27.5) for CSF polymerase chain reaction positivity. We found that the headache, fever, vomiting, and abnormal chest radiograph were the most common symptoms and diagnostic findings among tuberculous meningitis patients.

**Conclusions:**

Despite anti-tuberculosis treatment, adult tuberculous meningitis has very poor outcomes. The mortality rate of patients diagnosed in stage III or HIV co-infection increased significantly during treatment.

## Background

Tuberculosis, caused by *Mycobacterium tuberculosis* (MTB), remains one of the leading causes of infection-related mortality worldwide [1]. In 2017, an estimated 10 million incident cases of tuberculosis were recorded globally with approximately 1.57 million deaths [1]. Tuberculous meningitis is the most devastating presentation of disease with MTB [2], which accounts for approximately 1% of all cases of active tuberculosis, and 5 to 10% of extra-pulmonary tuberculosis cases [3, 4]. Tuberculous meningitis is especially common in children and those infected with human immunodeficiency virus (HIV), in whom outcomes are poor [2, 5].

Early diagnosis, prompt anti-tuberculosis treatment and corticosteroids are the main determinants of outcome in tuberculous meningitis [2]. However, early diagnosis of tuberculous meningitis remains a great challenge because symptoms such as fever, headache, vomiting and so on, are not specific. Since identification of acid-fast bacilli in the cerebrospinal fluid (CSF) and culture of MTB lack sensitivity, the diagnosis of tuberculous meningitis is often based on clinical suspicion combined with empirical decision making [3]. The disease severity is first classified into three stages according to the British Medical Research Council (BMRC) [6]. The following clinical stages are defined: stage I: fully conscious patient with no focal neurological deficits; stage II: there is altered sensorium but not to the degree of coma and minor focal neurological deficits such as a single cranial nerve palsy; stage III: marked alteration of level of consciousness, coma. With the introduction of the Glasgow Coma Scale (GCS) [7], this was modified as grade I (GCS 15; no focal neurological signs), grade II (GCS 11–14, or 15 with focal neurological signs), and grade III (GCS ≤10) disease [8]. This type of classification is useful to predict prognosis.

Without treatment, tuberculous meningitis leads to death. An effective treatment regimen recommended by the World Health Organization (WHO) consists of isoniazid, rifampicin, and pyrazinamide, usually with a fourth drug such as ethambutol or streptomycin, as first-line anti-tuberculosis drugs in patients with tuberculous meningitis [9, 10]. In addition to effective anti-tuberculosis treatment, adjuvant corticosteroid treatment is also recommended for tuberculous meningitis patients [2, 4, 9, 10].

There were many studies described the treatment outcome for tuberculous meningitis, but the results varied between studies due to inconsistent diagnostic criteria, treatment methods, study populations and settings. A previous systematic review of research showed that the prognosis of tuberculous meningitis in children are very poor, Especially for patients in stage III [5]. However, outcomes for adult patients have not been systematically reviewed.

Therefore, this systematic review and meta-analysis were performed to evaluate the prognosis of adult tuberculous meningitis. Our primary objective was to establish risks of death in adult tuberculous meningitis patients during treatment. Additionally, we reported the pooled frequencies of symptoms and diagnostic findings at presentation.

## Methods

This systematic review was conducted according to the Preferred Reporting Items for Systematic Reviews and Meta-Analyses (PRISMA) guidelines.

### Search strategy and selection criteria

We searched the Ovid MEDLINE, EMBASE, Cochrane Library and Web of Science databases to identify all relevant studies published up to May 8, 2018. The search terms were used as follows: “tuberculous meningitis” OR ((tubercul* OR tb) AND mening*) OR tuberculous meningitis.

Inclusion criteria were as follows: (1) original study; (2) reported in English; (3) described treatment regimens and outcomes, disaggregated outcomes for adult tuberculous meningitis; (4) including at least 10 adults, and less than 10% of patients lost-to-follow-up. Exclusion criteria were as follows: (1) studies of children < 14 years; (2) patients already included in another report. For duplicative or overlapping publications, the study with the largest sample size was included. Studies obtained from the literature search were checked by title and abstract. Relevant studies were examined in full text. Two authors (MG W and YX Z) independently screened all potentially relevant studies and tried to reach a consensus on all items. Any disagreements were assessed by a third author (XM L).

The diagnosis of tuberculous meningitis was based on clinical, CSF, radiological criteria and evidence of tuberculosis elsewhere [11]. Tuberculous meningitis was classified as “definite” if CSF smear was positive for AFB and/or culture positive for MTB, or positive for polymerase chain reaction for MTB, or AFB seen in the context of histological changes consistent with TB brain or spinal cord together with suggestive symptoms/signs and CSF changes, or visible meningitis (on autopsy) [11]. Tuberculous meningitis was termed as “probable” if total score of ≥12 when neuroimaging available or total score of ≥10 when neuroimaging unavailable. At least 2 points should either come from CSF or cerebral imaging criteria [11]. Tuberculous meningitis was classified as “possible” if total score of 6–11 when neuroimaging available, or total score of 6–9 when neuroimaging unavailable [11].

### Data extraction and definitions

Two independent authors (MG W and YX Z) extracted data from included studies using a standardized abstraction form, and a third (XM L) arbitrated discrepancies. The following data were extracted from each study: treatment outcomes, characteristics of studies and patients, and frequencies of symptoms and diagnostic results. Outcome indicators included death, neurological sequelae, and lost-to-follow-up (treatment abandonment or loss to follow-up). Survival is defined as being alive at the time of completion of treatment. Neurological sequelae are defined as any motor, sensory, cognitive, or hypothalamic impairment that emerged during the disease and continuous the treatment period.

### Quality assessment

The quality of individual studies was assessed with only high quality studies included for analysis. High quality studies were prospective cohort, retrospective consecutive cohort, or randomized control in design; reported a treatment duration at least 6 months, and follow-up of at least 6 months; reported basic demographic data; had less than 10% of patients lost-to-follow-up.

### Statistical analysis

Microsoft Excel (version 13.0) and R (version 3.5.1) software were used for data entry and analysis. The random effects model was used to generate summary effect. Logit transformation was used for all meta-analyses.

First, we pooled all studies to estimate the risk of death and the proportion of survivors in adult patients with tuberculous meningitis during treatment. To further explore the relationship between disease severity and treatment outcome, studies that stratified outcomes by BMRC or the modified BMRC disease stage were used to calculate the risk of death at different disease stages during treatment [6, 8].

Secondly, we also pooled the demographic characteristics of all patients, including the frequencies of symptoms and diagnostic results.

## Results

**Fig. 1:**
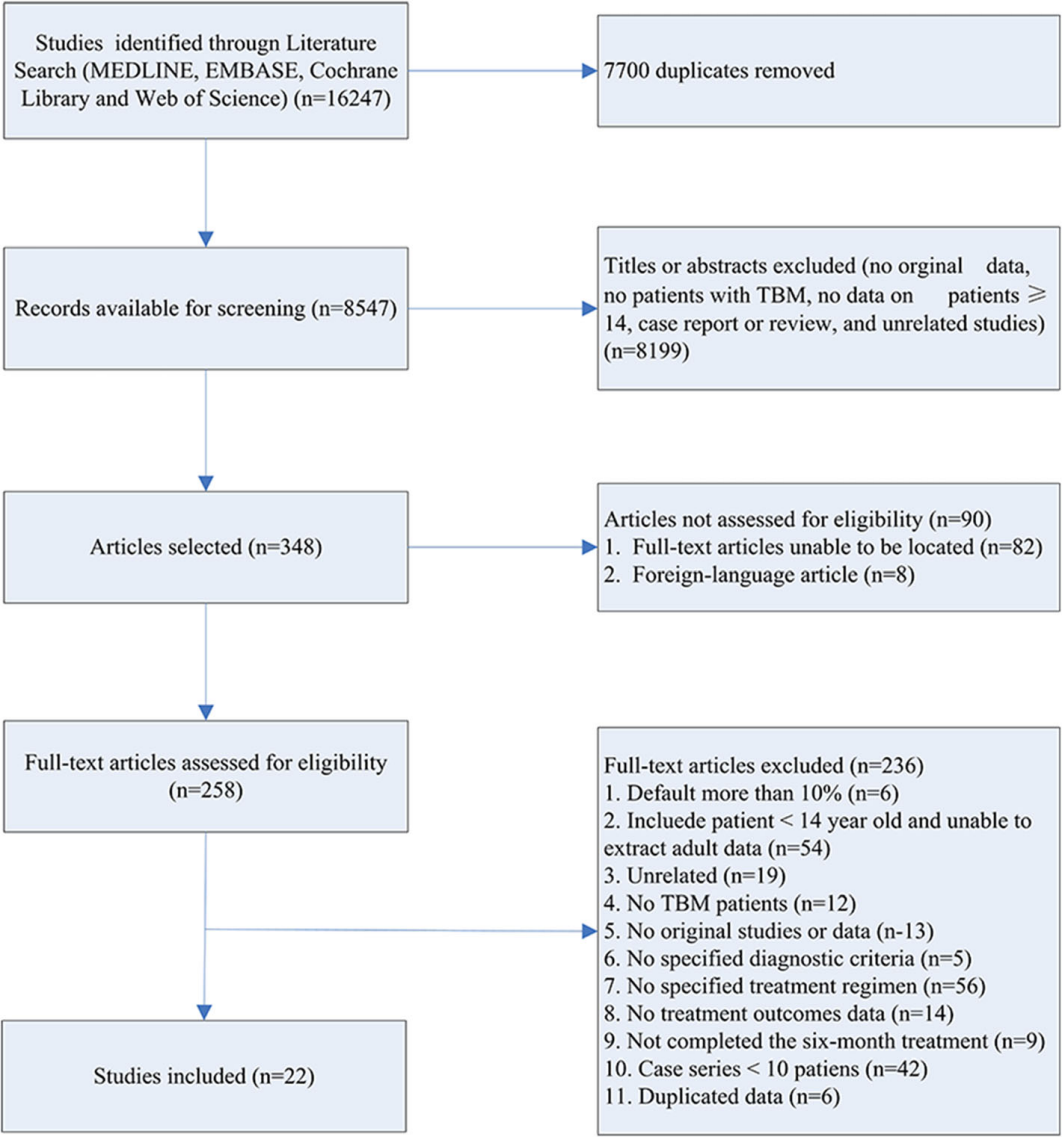
Flow diagram of included studies

**Fig. 2:**
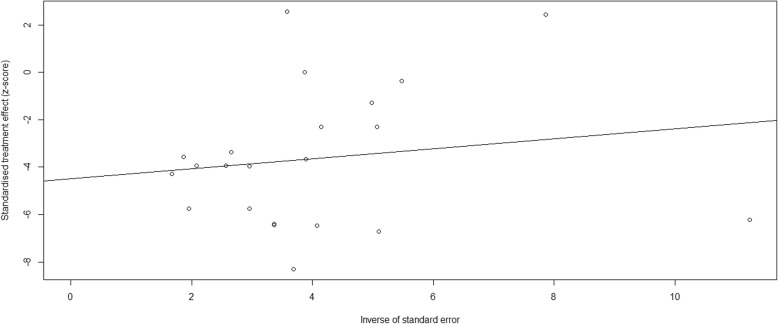
The Egger’s funnel plot of publication bias

A flow chart outlining our literature search is shown in Fig. 1. We identified 16,247 publications from our initial database search. After removal of repetitive studies, 8547 articles were screened by titles and abstracts. Of these, 348 articles were identified for full text review and 90 articles were not assessed for eligibility. Two hundred and thirty-six studies were removed prior to analysis, as shown in Fig. 1. Consequently, 22 articles were included in the systematic review and meta-analysis (Table 1) [12–33]. Publication bias was found by both Begg’s test and Egger’s test Fig. 2.

**Table 1:** Characteristics of included studies

Study	Study type	Location	Year of enrolment	Number of patients	Intensive phase		Continuation phase		Patients receiving corticosteroids	Follow-up (months)	Patients characteristics			
					Treatment	Duration (months)	Treatment	Duration (months)			Age (years)	Female	Lost-to-follow-up	HIV
Hosoglu et al (1998)	RC	Turkey	1985–1996	101	HRSZ (group 1); HRSE (group 2)	2–3	HR	≥ 6	NR	NR (≥ 9)	30.6 ± 13.0	40	5	NR
Katrak et al (2000)	PC	India	1992–1997	53	HRZE	≥ 6			NR	6	16–60	NR	0	22
Chan et al (2003)	RC	China	1997–2001	31	HRZE	3	HR	9	NR	12–60	≥ 14	17	NR	1
Thwaites et al (2004)	PC	Vietnam	2001–2003	545	HRZS (group 1); HRZE (group 2)	3	HRZ	6	Some	9	15–88	214	34	98
Cagatay et al (2004)	RC	Turkey	1991–2002	42	HRZE	2–3	HR	10	Some	12	16–60	28	NR	2
Torok et al (2008)	PC	Vietnam	2004–2005	58	HRZE (group 1); HRZES (group 2)	3	HRZ	6	NR	NR (≥ 9)	20–48	7	8	36/55
Hsu et al (2010)	RC	China	2000–2006	108	HRZ (group 1); HRZE (group 2)	≥ 6			Some	≥ 6	19–87 (54.9 ± 18.6)	37	NR	NR
Sinha et al (2010)	PC	India	2008–2009	101	HRZS	2	HR	7	All	6	14–85 (30 ± 13)	42	NR	0
Anuradha et al (2010)	PC	India	2008–2009	100	HRZE	2	HR	4	All	6	> 14 (30 ± 13)	41	NR	0
Sharma et al (2011)	RC	India	2008–2010	158	HRZS	2	HR	7	All	≥ 9	> 14 (31.95 ± 13.96)	91	NR	0
Torok et al (2011)	RCT	Vietnam	2005–2007	253	HRZE (group 1); HRZES (group 2)	3	HR	6	All	≥ 9	≥ 15	25	NR	253
Marais et al (2011)	RC	South Africa	2009	120	HRZE (group 1); HRZES (group 2)	2–3	HR (group 1); HRE (group 2)	≥ 4 (group 1); ≥ 5 (group 2)	Some	≥ 6	≥ 18	60	12	106/114
Raut et al (2013)	PC	India	2010–2012	80	HRZS	2	HR	7	NR	6	14–68	37	NR	0
Ruslami et al (2013)	RCT	Indonesia	2010–2011	60	HRZE (group1); HRZMfx (group 2)	2	HR	4	All	≥ 6	> 14	27	NR	7
Imam et al (2014)	RC	Qatar	2006–2012	80	HRZE	≥ 6			Some	NR (≥ 6)	≥ 18	15	1	NR
Iype et al (2014)	PC	India	2010–2011	45	HRZS (group 1); HREZ (group 2)	2–3	HR	7	NR	NR (≥ 9)	14–61	21	2	NR
Chen et al (2014)	RC	China	2002–2009	38	HRZE	2	HRE	10月16日	Some	NR (12–18)	> 18	133	3	2
Jha et al (2015)	PC	India	2012–2014	118	HRZS	2	HR	7	All	6	≥ 14	61	NR	0
Misra et al (2015)	RC	India	2010–2014	74	HRZE	6	HR	12	ALL	NR (≥ 18)	≥ 14	28	NR	NR
Tai et al (2016)	PC	Malaysia	2009–2014	41	HRZE	2	HR	10月16日	Some	NR (12–18)	35.0 ± 13.7	19	NR	0
Li et al (2017)	RC	China	2012–2015	156	HRZE	2–4	HR	6月12日	All	≥ 8	16–82	69	14	NR
Raberahona et al (2017)	RC	Madagascar	2007–2014	75	HRZE (group 1); HRZES (group 2)	2	HR (group 1); HRZE (group 2)	6 (before 2012); 4 (after 2012)	NR	NR (≥ 6)	≥ 18 (35.4 ± 12.7)	33	NR	3

(Abbreviations: *HIV* human immunodeficiency virus, *RC* retrospective cohort, *PC* prospective cohort, *RCT* randomized controlled trial, *H* isoniazid, *R* rifampicin, *Z* pyrazinamide, *E* ethambutol, *S* streptomycin, *Mfx* moxifloxacin, *NR* not reported)

The basic characteristics of the included studies are shown in Table 1. Of the 22 eligible studies, 11 were retrospective chart reviews, 9 were prospective cohorts, and two were randomized controlled trial. The study periods ranged from 1998 to 2017. The study populations of these studies came from nine countries. Seventeen studies were conducted in countries currently on the WHO list of countries with high TB burden [13–25, 27–29, 31]. The majority of patients were male (61.2%). In studies with available data, 10.6% (95% CI: 4.2–24.6) of patients were infected with HIV.

**Fig. 3:**
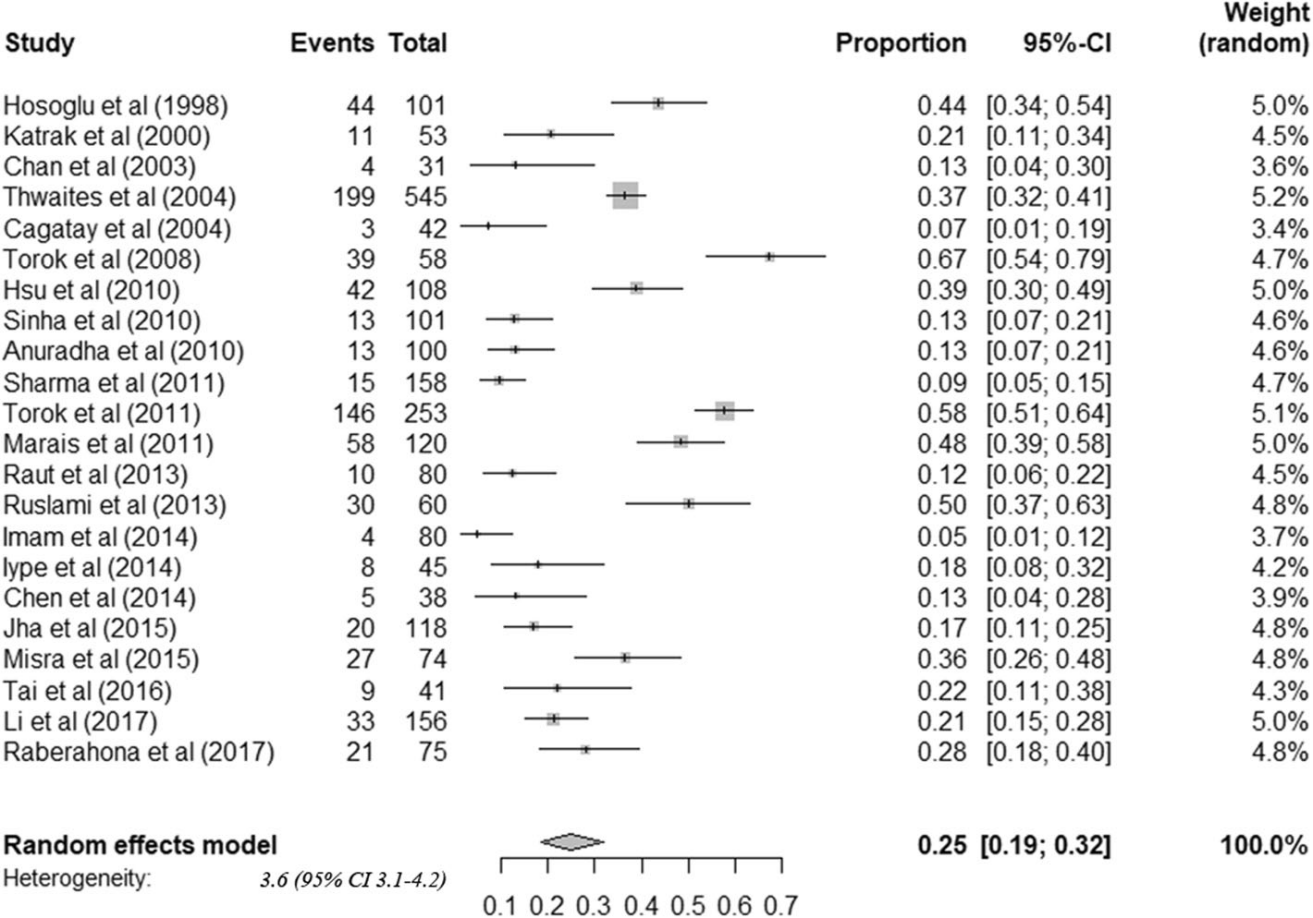
Frequency of death among tuberculous meningitis in adults

**Fig. 4:**
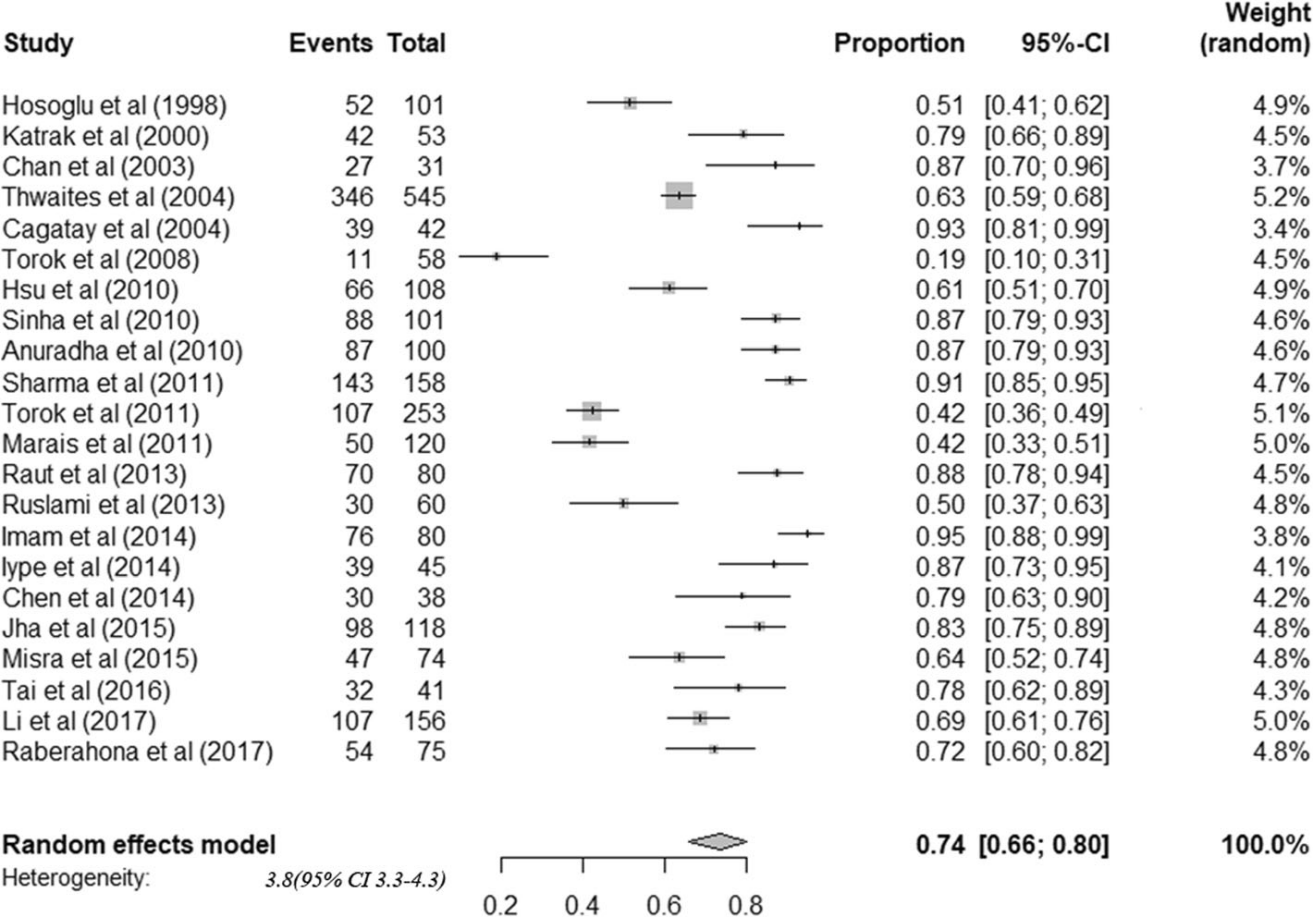
Frequency of neurological sequelae among survivors

The 22 cohorts included data from 2437 patients. All tuberculous meningitis patients received anti-tuberculosis treatment. Among adult tuberculous meningitis patients, risk of death was 24.7% (95%CI: 18.7–31.9) (Fig. 3). Among survivors, risk of neurological sequelae was 50.9% (95%CI: 40.2–61.5) (Fig. 4). By summarizing the results of 17 studies that stratified treatment outcomes according to disease stages, we found that the risk of death was significantly higher among patients diagnosed in stage III (64.8%) than stage I (17.5%) or II (28.5%) (Table 2). Moreover, patients co-infected with HIV were found to have higher mortality (HIV positive: 53.4% (42.4–64.1), HIV negative: 17.5% (12.1–24.7)) (Table 2). Considerable heterogeneity was observed for all outcomes.

**Table 2:** Subgroup analyses of deaths

	Number of studies	Number of deaths	Number of patients assessed	Proportion of death (95%CI)	Heterogeneity (95%CI)
Study type					
Prospective cohort	9	322	1141	22.4%(14.1–33.8)	3.4(2.7–4.4)
Retrospective cohort	11	205	983	22.0%(14.5–31.8)	3.1(2.5–4.0)
BMRC stage					
Stage I	7	60	356	17.5%(8.9–31.7)	1.8(1.2–2.7)
Stage II	7	157	513	28.5%(20.6–37.9)	1.7(1.2–2.6)
Stage III	7	207	318	64.8%(51.5–76.1)	1.9(1.3–2.8)
HIV infection					
HIV positive	7	301	547	53.4%(42.4–64.1)	2.1(1.4–3.0)
HIV negative	11	257	1264	17.5%(12.1–24.7)	2.7(2.1–3.5)
Treatment duration					
At least 6 months	9	256	853	27.9%(19.7–38.8)	3.1(2.4–4.0)
At least 9 months	13	498	1584	22.4%(14.5–32.8)	4.0(3.4–4.8)
Treatment with streptomycin					
Yes	5	102	558	17.1(8.5–31.6)	3.5(2.5–4.9)
No	11	181	783	20.3%(13.6–29.2)	2.6(2.0–3.3)

(Abbreviations: *BMRC* the British Medical Research Council, *HIV* human immunodeficiency virus)

Subgroup analyses were conducted to investigate the sources of heterogeneity, including study type, BMRC disease stage, HIV infection, treatment duration, and the use of streptomycin (Table 2). Unfortunately, we can not fully explain the heterogeneity of the research.

The population characteristics are summarized in Table 3. The most common features of patients were fever, headache, vomiting, weight-loss, abnormal chest radiograph and basilar enhancement (Table 3). Among 17 studies stratified patients by disease severity, 24.9% (21.1–29.1%) of patients were in stage I, 46.9% (41.4–52.4%) in stage II and 26.4% (20.7–32.9%) in stage III. Nearly 38.8% of the patients were diagnosed as definite tuberculous meningitis. The frequency of CSF acid-fast-bacilli smear positivity was 10.0% (5.5–17.6), the frequency of CSF culture positivity for MTB was 23.8% (15.2–35.3), and the frequency of CSF polymerase chain reaction positivity for MTB was 22.3% (17.8–27.5) of patients. All these pooled proportions showed significant between study heterogeneity.

**Table 3:** Frequency of symptoms and diagnostic findings at admission

	Number of studies	Number of patients with characteristics	Number of patients assessed	Proportion (95%CI)	Heterogeneity (95%CI)
Characteristics					
Females	21	925	2384	39.9%(34.0–46.0)	2.8(2.3–3.3)
Previous BCG vaccination	4	117	286	44.8%(22.4–69.5)	3.7(2.6–5.4)
Known tuberculosis contact	4	98	298	21.6%(3.8–65.5)	5.5(4.1–7.4)
Lost-to-follow-up	9	79	1196	7.3%(5.3–10.0)	1.3(1.0–1.9)
HIV positive	16	530	1864	10.6%(4.2–24.6)	4.3(3.7–5.0)
BMRC stage					
Stage I	17	534	2041	24.9% (21.1–29.1)	1.9 (1.5–2.5)
Stage II	17	927	2041	46.9%(41.4–52.4)	2.3(1.9–2.9)
Stage III	17	562	2041	26.4%(20.7–32.9)	2.9(2.4–3.5)
Diagnostic					
Possible	11	363	1399	29.0%(21.0–38.5)	3.2(2.6–4.1)
Probable	13	583	1565	34.7%(27.8–42.3)	2.8(2.2–3.5)
Definite	14	611	1643	38.9%(32.1–46.1)	2.6(2.0–3.2)
Symptoms					
Fever	14	1013	1126	89.5%(84.2–93.2)	2.1(1.6–2.7)
Headache	16	1089	1306	85.9%(77.9–91.4)	3.1(2.5–3.7)
Seisures	15	260	1369	18.5%(14.2–23.7)	2.2(1.8–2.8)
Vomiting	11	511	932	54%(40.3–67.1)	3.8(3.1–4.7)
Weight loss	8	319	611	47.9%(24.5–72.2)	3.9(3.1–5.0)
Radiological findings					
Abnormal chest radiogragh	9	465	781	54.5%(42.2–66.3)	3.1(2.4–4.0)
Basilar enhancement	14	565	1100	49.5%(28.8–70.5)	5.3(4.5–6.1)
Hydrocephalus	18	511	1461	35.2%(27.9–43.2)	2.8(2.4–3.4)
Infarction	13	273	1221	23.2%(18.3–29.0)	2.2(1.7–2.8)
Tuberculoma	16	359	1326	22.4%(16.9–29.0)	2.6(2.1–3.2)
Diagnostic testing					
Positive culture for *Mycobacterium tuberculosis* in CSF	18	588	1514	23.8%(15.2–35.3)	3.9(3.3–4.6)
Positive smear for acid-fast bacilli in CSF	14	166	1177	10.0%(5.5–17.6)	3.0(2.5–3.7)
Positive polymerase chain reaction for Mycobacterium tuberculosis in CSF	10	187	978	22.3%(17.8–27.5)	1.7(1.2–2.4)
Mycobacterium tuberculosis isolated from site other than CSF	4	70	404	14.7%(7.0–28.1)	2.7(1.8–4.3)

(Abbreviations: *BMRC* the British Medical Research Council, *CSF* cerebrospinal fluid)

## Discussion

This systematic review and meta-analysis estimated the treatment outcomes among adult tuberculous meningitis patients. The findings suggested that the treatment outcomes for adult patients with tuberculous meningitis are poor. In addition, our results show that the treatment outcomes are related to the BMRC grades and HIV co-infection.

To the best of our knowledge, this is the first meta-analysis to access the treatment outcome of tuberculous meningitis among adults. The results showed that 24% of tuberculous meningitis died during the treatment. More importantly, our subgroup analyses indicated that mortality increased with the severity of the disease. The more serious the disease was, the worse the treatment outcome was. Furthermore, tuberculous meningitis patients who were HIV positive had higher mortality. According to the WHO, 9.2% new tuberculosis cases were HIV positive (0.92 million) and 0.3 million deaths that were attributed to co-infection in 2017 [1]. Our study found that approximately 10.4% of patients with tuberculous meningitis were HIV positive. It has been reported that tuberculosis patents co-infected with HIV were more likely to have poor treatment outcome and death [34, 35]. Consisted with those studies, our results showed half of HIV-positive tuberculous meningitis patients died during the treatment, which was significantly higher than HIV negative patients (17.4%).

Early diagnosis of tuberculous meningitis is a great challenge for early treatment as there are limitations in the current widely used methods, such as the low sensitivity of the acid-fast bacilli smear and the long turn-around time of mycobacterial culture [36]. In this study, the definite diagnostic rate was 38.9%. Recently, rapid, sensitive and highly specific molecular detection methods have been favored [1, 37, 38]. Nearly 22.3% patients were positive for CSF polymerase chain reaction for MTB in this study. CSF molecular diagnostic methods (nucleic acid amplification tests) have previously been included in diagnostic criteria for tuberculous meningitis [37, 38]. While we found fever, headache, vomiting and weight-loss were the most common symptoms among tuberculous meningitis patients, these nonspecific clinical presentations are and thus may contribute to delayed diagnosis [2]. Hence, clinicians should be vigilant against the disease, and suspected patients should be treated with anti-tuberculosis drug based on rich clinical experience without waiting for confirmatory testing.

Effective anti-tuberculosis therapy is crucial for the treatment outcome of tuberculous meningitis. We excluded 56 of 258 full-text articles that do not specify treatment regimens, and 9 for lack of follow-up time or incomplete anti-tuberculosis treatment. As recommended by the WHO, all populations included in this study were treated for at least 2 months of intensive phase treatment (consisting of isoniazid, rifampicin, pyrazinamide, and ethambutol or streptomycin) [4, 9], followed by a continuation phase (consisting of isoniazid, rifampicin). Our results showed that the mortality of tuberculous meningitis was nearly in patients treated with streptomycin (17.1%) compared with ethambutol (20.3%). Which means neither the use streptomycin or not has no significant effect on treatment outcome. While the studies utilized the same regimens for tuberculous meningitis, the treatment durations were varied between studies [4, 9, 39]. In this systematic review, only those who completed anti-tuberculosis treatment for at least 6 months were included. This study found that mortality was high for both treatment at least 6 months and 9 months. Duo to the high mortality and sequelae of tuberculous meningitis, we believe that the course of treatment should be individualized.

Substantial heterogeneity was found between studies. Although we detected subgroup analysis based on the characteristics of the included studies, we still can not fully explain the source of heterogeneity. Although we failed to determine the source of heterogeneity, the following factors may related to heterogeneity. First, the different study designs of included studies, which might have led to the heterogeneity of the results. However, the similar result detected in prospective cohort study subgroups, reinforced our conclusion. Since, only two randomized controlled studies were included, the subgroup could not be assessed. Second, the study publication years ranged from 1998 to 2017, and the enrollment in some studies occurred before 1998. Although there had not been dramatic changes in how tuberculous meningitis was treated, the study period may be a cause of heterogeneity. Third, the severity of the disease in each study was different, which might be another factor leading to heterogeneity.

Our study has some limitations. First, in this meta-analysis, we only included studies published in English, eight studies reported in other language were not assessed for full-text reading. Second, we excluded studies with more than 10% of patients lost-to-follow-up. Although we did not include these studies, the treatment outcomes of patients with tuberculous meningitis were consistent with our results [40, 41]. Third, the high mortality rate of tuberculous meningitis may be associated with several factors, such as stage of tuberculous meningitis disease, HIV co-infection, treatment delay, drug resistance, corticosteroid use or the incidence of stroke. Previous studies demonstrated that HIV co-infection, drug resistance, advanced stage of tuberculous meningitis at admission and the incidence of stroke were associated with poor outcome and mortality among tuberculous meningitis patients [10, 17, 20]. Although we were unable to assess all of these associations, the high mortality in stage III and HIV co-infection among tuberculous meningitis patients, also suggested that the severity of tuberculous meningitis and HIV co-infection are associated with treatment outcome. Forth, we did not evaluate the effect of corticosteroid use on the treatment outcomes of patients with tuberculous meningitis. However, previous randomized controlled trials provided us evidence that tuberculous meningitis patients will benefit from corticosteroid use [15, 42]. Fifth, substantial publication bias was found by both Begg’s test and Egger’s test. The following reasons may lead to publication bias. Language bias, we only included studies published in English language in this study. Methodological quality differences, smaller studies were conducted and analyzed with less methodological rigour than larger studies. The sample size of the included studies ranged from 31 to 545, and we also found that the sample sizes of 12 studies were less than 100. Moreover, we only included studies had less than 10% of patient lost-to-follow-up, which may be another cause of publish bias. Since, there is a certain publication bias in this study, further prospective cohort and randomized controlled trial studies are needed.

## Conclusion

Tuberculosis remains a major global health problem. Treatment outcomes for adult tuberculous meningitis are very poor, especially for patients diagnosed in stage III or HIV co-infection. The early diagnosis of tuberculous meningitis is hampered by the low sensitivity of cerebrospinal fluid microscopy and the slow growth of MTB in conventional culture systems. Rapid, sensitive and specific molecular detection methods should be widely used in the diagnosis of tuberculous meningitis. Effective anti-tuberculosis and adjunctive corticosteroid therapy is crucial for the treatment outcome of tuberculous meningitis.

## Data Availability

The datasets used and/or analysed during the current study are available from the corresponding author on reasonable request.

## Abbreviations

BMRC: British Medical Research Council
CSF: Cerebrospinal fluid
E: Ethambuto
GCS: Glasgow Coma Scale
H: Isoniazid
HIV: Human immunodeficiency virus
Mfx: Moxifloxacin
MTB: *Mycobacterium tuberculosis*
PC: Prospective cohort
PRISMA: Preferred Reporting Items for Systematic Reviews and Meta-Analyses
R: Rifampicin
RC: Retrospective cohort
RCT: Randomized controlled trial
S: Streptomycin
WHO: World Health Organization
Z: Pyrazinamide
